# Evaluating the Neuroprotective Potential of Caffeinated Coffee in the Context of Aluminum-Induced Neurotoxicity: Insights from a PC12 Cell Culture Model

**DOI:** 10.3390/antiox13030342

**Published:** 2024-03-13

**Authors:** Kamil Rodak, Dorota Bęben, Monika Birska, Oliwia Siwiela, Izabela Kokot, Helena Moreira, Anna Radajewska, Anna Szyjka, Ewa Maria Kratz

**Affiliations:** 1 Department of Laboratory Diagnostics, Division of Laboratory Diagnostics, Faculty of Pharmacy, Wroclaw Medical University, Borowska Street 211A, 50-556 Wroclaw, Poland; izabela.kokot@umw.edu.pl; 2 Student Research Club, “Biomarkers in Medical Diagnostics”, Department of Laboratory Diagnostics, Division of Laboratory Diagnostics, Faculty of Pharmacy, Wroclaw Medical University, Borowska Street 211A, 50-556 Wroclaw, Poland; 3 Student Research Club of Flow Cytometry and Biomedical Research, Department of Basic Medical Sciences and Immunology, Faculty of Pharmacy, Wroclaw Medical University, Borowska Street 211, 50-556 Wroclaw, Poland; dorota.biegas@student.umw.edu.pl (D.B.); monika.birska@student.umw.edu.pl (M.B.); oliwia.siwiela@student.umw.edu.pl (O.S.); 4 Department of Basic Medical Sciences and Immunology, Faculty of Pharmacy, Wroclaw Medical University, Borowska Street 211, 50-556 Wroclaw, Poland; helena.moreira@umw.edu.pl (H.M.); anna.szyjka@umw.edu.pl (A.S.); 5 Department of Medical Laboratory Diagnostics, Division of Clinical Chemistry and Laboratory Hematology, Faculty of Pharmacy, Wroclaw Medical University, Borowska Street 211A, 50-556 Wroclaw, Poland; a.radajewska@umw.edu.pl

**Keywords:** neurotoxicity, neurodegeneration, neurodegenerative diseases, oxidative stress, aluminum, coffee, caffeine

## Abstract

Exposure to aluminum (Al) and its compounds is an environmental factor that induces neurotoxicity, partially through oxidative stress, potentially leading to the development of neurodegenerative diseases. Components of the diet, such as caffeinated coffee, may play a significant role in preventing these diseases. In the present study, an experimental model of PC12 cells (rat pheochromocytoma tumor cells) was developed to investigate the influence of caffeine and caffeinated coffee on neurotoxicity induced by Al compounds and/or oxidative stress. For the induction of neurotoxicity, aluminum maltolate (Almal) and H_2_O_2_ were used. The present study demonstrates that 100 μM Almal reduced cell survival, while caffeinated coffee with caffeine concentrations of 5 μg/mL and 80 μg/mL reversed this effect, resulting in a higher than fivefold increase in PC12 cell survival. However, despite the observed antioxidant properties typical for caffeine and caffeinated coffee, it is unlikely that they are the key factors contributing to cell protection against neurotoxicity induced by both oxidative stress and Al exposure. Moreover, the present study reveals that for coffee to exert its effects, it is possible that Al must first activate certain mechanisms within the cell. Therefore, various signaling pathways are discussed, and modifications of these pathways might significantly decrease the risk of Al-induced neurotoxicity.

## 1. Introduction

**Figure 1:**
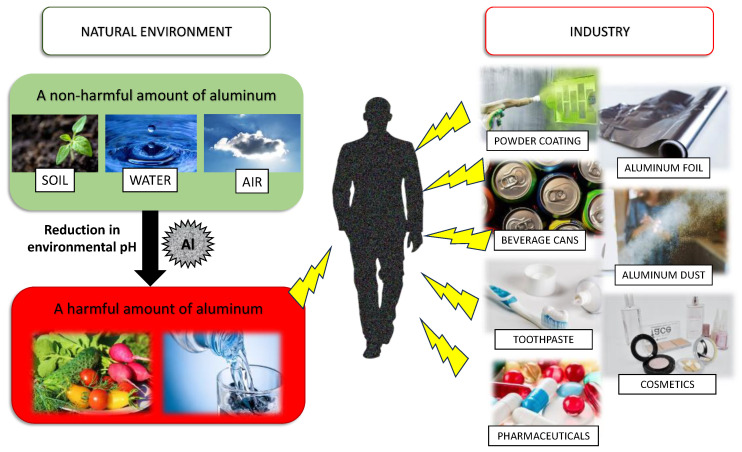
Exemplary sources of aluminum exposure.

Aluminum (Al) is distinguished as one of the most abundant metallic elements and occupies the third position in terms of prevalence within the Earth’s crust [1]. While natural phenomena and the influence of acidic precipitation play a role in the dispersion of Al throughout the environment, thereby contributing to its inherent presence in food and water sources, the expanding industrialization of the world has caused increased Al levels in humans’ surroundings. Al is currently integrated into a diverse range of consumer products readily available to the general public, including food, drinking water, processed foods, cosmetics, toothpaste, antiperspirants, as well as a multitude of medical formulations and pharmaceuticals (Figure 1) [2]. Excluding occupational exposure, it is important to note that the diet constitutes the primary source, accounting for 95% of the total Al in the human body [3]. Consequently, the widespread utilization of Al in the above-mentioned commodities results in human exposure to this element, which is nearly inevitable.

Although the absorption of Al in its insoluble form into the human body remains at a low level (ranging from 0.01% to 1%) [4], in other chemical forms, such as citrate, lactate, gluconate, maltolate, and oxalate, its absorption is comparatively higher [5]. Importantly, Al has the capability to traverse the blood–brain barrier. As a result, excessive exposure to Al may cause neurotoxicity and lead to the development of neurodegenerative diseases, i.e., Alzheimer’s disease (AD) and Parkinson’s disease (PD) [6,7]. The etiology of neurodegenerative diseases is diverse and often not fully understood. Frequently, it is associated with the accumulation of pathological proteins in the brain, such as amyloid beta (Aβ) in AD or α-synuclein in PD [8,9]. It is widely recognized that metal ions, particularly Al, possess the capacity to engage with diverse proteins, provoking conformational alterations. Biochemical transformations, such as protein misfolding, aggregation, or oligomerization, may contribute significantly to the pathophysiology of neurodegenerative diseases. Numerous authors have reported that Al appears to stimulate the expression of the amyloid precursor protein (APP), elevate the levels of β-40 and β-42 fragments of amyloid within the brain, and facilitate the aggregation of Aβ [8,10,11]. Nonetheless, distinct in vivo approaches have failed to replicate these outcomes regarding the Aβ pathway [12,13]. Moreover, it has been ascertained that Al plays a role in protein phosphorylation as well as protein aggregation, notably the tau protein, which is a characteristic feature of AD [14]. Furthermore, Al’s influence on neurotransmission has been substantiated, primarily through its impact on the cholinergic system (particularly the activity of the acetylcholinesterase enzyme (AChE)), which may be also implicated in the pathogenesis of AD [15]. Notwithstanding the considerable scientific endeavors and current comprehension of the mechanisms underlying Al neurotoxicity (reviewed by Skalny et al. [16]), a unanimous consensus regarding the precise involvement of Al in neurodegenerative diseases remains elusive [17,18]. However, it can be confidently asserted that an association between these factors exists, as substantiated by epidemiological studies [19].

Nonetheless, another factor intricately associated with neurotoxicity and neurodegenerative diseases is oxidative stress manifesting by an imbalance between the production of free radicals, such as reactive oxygen species (ROS), and the body’s ability to counteract or neutralize their harmful effects through the action of antioxidants [20,21]. This imbalance results in the accumulation of ROS surpassing the body’s defense mechanisms, leading to potential damage of cells, proteins, lipids, and DNA [21]. Oxidative stress is also recognized as one of the significant causes of neural tissue damage [22]. Hence, it might be essential to focus on the association between Al exposure and oxidative stress for cognitive well-being. Some researchers have stated that Al exhibits pronounced prooxidant characteristics [23,24]. Several mechanisms may be responsible for this phenomenon. One of them is the adverse impact of Al on iron (Fe) homeostasis, as the interaction between these two elements leads to the formation of a labile form of Fe from Fe-containing enzymes and proteins [25]. Consequently, this results in an elevated pool of free Fe in the human body, leading to increased production of ROS associated with the Fenton reaction [25,26]. Another mechanism behind the prooxidative activity of Al may involve the generation of superoxide Al^3+^ semi-reduced radicals [23,27]. These radicals reduce the activity of the body’s antioxidant system, particularly enzymes such as catalase, superoxide dismutase, and glutathione peroxidase [28,29]. This results in heightened vulnerability of tissues and cells, including neural cells, to oxidative damage [29].

Due to the widespread consumption of caffeinated coffee, which is a rich source of antioxidants such as polyphenols, there has been growing interest in exploring whether caffeinated coffee and caffeine itself might exert a protective effect against Al-induced neurotoxicity [30,31,32]. The principal constituent of coffee is caffeine, which has been ascribed to various health-promoting properties [33]. Within the human body, caffeine primarily exerts its effects through adenosine receptors (ARs), the largest amount of which is found in the nervous system [33]. Through interactions with these receptors, caffeine influences brain functions and even provides protection against processes associated with the development of numerous diseases, including AD [34]. Li et al. [35] elucidated a mechanism of caffeine action against Aβ generation that involves the suppression of amyloid beta protein precursor (AβPP) by impeding AβPP interaction with adenosine receptors—A_3_Rs. Furthermore, the upregulation of adenosine receptors A_1_R and A_2A_R is observed in AD [36]. Moreover, some epidemiological studies confirmed that the consumption of caffeinated coffee reduces the risk of AD development. Eskelinen et al. [37], in their long-term study, exhibited that the intake of 3–5 cups of caffeinated coffee per day decreases the susceptibility to the development of this disease by as much as 62–70%. A different study conducted by Liu et al. [38], involving a cohort of 187,499 men and 130,761 women, revealed that increased consumption of caffeinated coffee was linked to a decreased risk of PD in a dose-dependent manner.

Considering the association among aluminum exposure, its neurotoxicity, and the onset of neurodegenerative diseases as well as its association with oxidative stress and the antioxidant properties of caffeine and caffeinated coffee along with their favorable impact on reducing the risk of neurodegenerative diseases an experimental model that aims to investigate the influence of caffeinated coffee and caffeine on neurotoxicity induced by Al compounds and/or oxidative stress was developed in the present study. For this purpose, PC12 cells (rat pheochromocytoma tumor cells) were employed, as they can serve as an in vitro model for neurodegenerative processes [39,40]. Based on literature sources [41], in the present study, aluminum maltolate (Almal) and H_2_O_2_ were utilized for the induction of neurotoxicity. In our research, we wanted to evaluate whether caffeinated coffee and/or caffeine possess neuroprotective properties under Al-induced neurotoxicity and, if so, whether this protective activity is associated with the reduction of oxidative stress. The present study also aimed to investigate whether caffeinated coffee/caffeine prevents the toxic effects of oxidative stress by reducing the level of ROS when hydrogen peroxide is added. Additionally, we aimed to determine whether the presence of oxidative stress and exposure to Almal yields a combined effect on cells that is more challenging to mitigate by caffeinated coffee or caffeine when compared to their individual effects. To the best of our knowledge, this is the only study that tested and illustrated the impact of caffeinated coffee on PC12 cells treated with Almal and considered all examined substances together.

## 2. Materials and Methods

### 2.1. Chemicals and Reagents

Anhydrous powdered caffeine was purchased from CristalChem (catalog no: 58-08-2, Poznań, Poland). Coffee (100% Arabica instant coffee, freeze-dried) contained 72 mg of caffeine in 1.8 g of instant coffee (as stated by the manufacturer) was provided by Tchibo (product reference No: tcs81037, Epsom, UK). Aluminum chloride hexahydrate (AlCl_3_ × 6H_2_O) (catalog no: 237078), 3-hydroxy-2-methyl-4-pyrone (catalog no: H43407), gentamicin (catalog no: G1397), MTT [3-(4,5-dimethyl-2-thiazolyl)-2,5-diphenyl-2H-tetrazolium bromide] (catalog No: M5655), DCF-DA (2,7-dichlorofluorescin diacetate) (catalog no: D6883) and Minimum Essential Medium (MEM) (catalog No: 51412C) were purchased from Sigma-Aldrich (St. Louis, MO, USA). Hydrogen peroxide (catalog no: 363-118851934) was purchased from Alchem (Wroclaw, Poland). RPMI-1640 medium (catalog no: 11875101), donor horse serum (DHS) (catalog no: 16050122), amphotericin B fungizone (catalog no: 15290018), and TrypLE^TM^ Express Enzyme (catalog no: 12604039) were purchased from ThermoFisher (Waltham, MA, USA). Fetal bovine serum (FBS) (catalog no: 04-007-1A) was from BI Biological Industries (Beit Haemek, Israel).

### 2.2. Reagent Preparation

#### 2.2.1. Coffee and Caffeine Solutions

Aqueous solutions of caffeine and caffeinated coffee were prepared by dissolving the respective quantities of caffeine and caffeinated coffee in water to achieve stock solutions with a caffeine concentration of 5 mg/mL (accordingly, 500 mg of caffeine and 12.5 g of coffee were dissolved in 100 mL of water). Further dilution was prepared directly in cell culture media in the wells of the plate to obtain final concentrations of caffeine at 5 μg/mL, 80 μg/mL, and 320 μg/mL.

#### 2.2.2. Aluminum Maltolate Solution

Aluminum maltolate was prepared according to the method of Bertholf et al. [42]. Here, 20 mM aqueous solution of aluminum chloride hexahydrate (AlCl_3_ × 6H_2_O) (pH = 3) and 20 mM maltol solution were utilized. Maltol solution was obtained by solubilizing 3-hydroxy-2-methyl-4-pyrone in 0.1 M phosphate-buffered saline (PBS) with mild heating, and the pH was adjusted to 7.4 using 1 M NaOH. The Almal stock mixture was made by combining equal volumes of AlCl_3_ × 6H_2_O and maltol, obtaining a final concentration of 10 mM for each component. The pH of the solution was adjusted to 7 using 1 M NaOH. A further dilution was prepared directly in cell culture media in the wells of the plate to obtain final concentrations of 50 μM and 100 μM.

### 2.3. Cell Culture Conditions

The PC12 cell line was obtained from the American Type Culture Collection (ATCC, Manassas, VA, USA). The cell line was derived from the rat adrenal pheochromocytoma tumor. The PC12 cells were cultured in flasks in RPMI-1640 medium enriched with 10% DHS, 10% FBS, 50 μg/mL gentamicin, and 250 μg/mL amphotericin B fungizone. The cells were passaged every three days using TrypLE^TM^ Express Enzyme. The cell cultures were grown under optimal conditions (37 °C in 95% humidity and 5% CO_2_).

**Figure 2:**
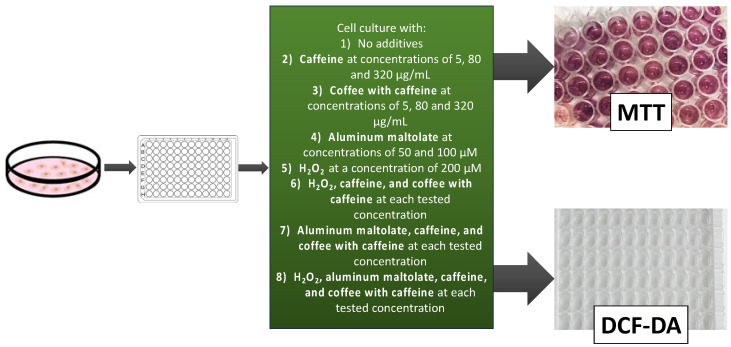
Schematic representation of the experimental procedure.

For experimental purposes, the PC12 cells were detached from the culture flask using TrypLE^TM^ Express Enzyme and transferred to a 96-well plate at an optimal seeding density of 5 × 10^4^ cells/200 µL per well [43]. To promote optimal cell adhesion to the plate bottom, an overnight incubation was performed. Then, the culture medium was replaced with fresh medium containing varying concentrations of the tested substances. Specifically, PC12 cells were exposed to caffeine (at concentrations of 5, 80, and 320 µg/mL), coffee with caffeine (at concentrations of 5, 80, and 320 µg/mL), Almal (at concentrations of 50 and 100 µM), H_2_O_2_ (at a concentration of 200 µM), or specific combinations of these substances for 24 h under optimal conditions (37 °C in 95% humidity and 5% CO_2_). The concentrations of caffeine were selected based on information available in the literature [44,45]. For each experimental configuration, four replicates were performed on a plate. After incubation with the tested substances, the medium was aspirated, and the cells were rinsed with PBS in order to minimize interferences between tested substances and reagents used in MTT and DCF-DA assays. Then, the MTT assay or DCF-DA assay was performed. The scheme of the experimental procedure is shown in Figure 2.

### 2.4. Cell Viability Assay

Cell viability was assessed using the MTT assay. MTT [3-(4,5-dimethyl-2-thiazolyl)-2,5-diphenyl-2H-tetrazolium bromide] is a yellow tetrazolium salt that is enzymatically reduced within metabolically active mitochondria to form an insoluble formazan compound. After 24 h of cell incubation with the tested substances, the medium containing these compounds was removed from the wells, and the cells were washed three times with PBS. Subsequently, a 1 mg/mL MTT solution in MEM was added to each well, and further incubation was carried out for two hours in the dark at 37 °C. Then, the MTT solution was aspirated, and isopropanol was added to each well. The cells were incubated for an additional 30 min in the dark at room temperature. Then, the absorbance was measured at 570 nm using the Varioskan LUX microplate reader (Biotek, Winooski, VT, USA).

Cell viability was calculated using the following equation:

$$\%\;\mathrm{Viability}=\frac{\mathrm{Absorbance}\;\mathrm{of}\;\mathrm{the}\;\mathrm{tested}\;\mathrm{cells}}{\mathrm{Absorbance}\;\mathrm{of}\;\mathrm{the}\;\mathrm{control}\;\mathrm{cells}}\times 100\%$$

Tested cells: cells incubated in the presence of the tested substances, control cells: cells incubated without any additives or with one tested substance (as presented in the descriptions of the relevant figures).

### 2.5. DCF-DA Assay

Antioxidant properties were assessed using the DCF-DA assay. Before each experiment, the DCF-DA solution was freshly prepared by dissolving 4 mg of 2′,7′-dichlorofluorescein diacetate in 820 µL of 96% ethanol, obtaining a 10 mM stock solution. Subsequently, the above solution was dissolved in MEM to yield a concentration of 1 mM. After 24 h of cell incubation with the tested substances, the well contents were aspirated. Cells were rinsed with PBS, and DCF-DA solution was added to obtain a concentration of 20 µM (as reported by Ng and Ooi [46]) for 1 h (incubation in the dark, at 37 °C). Then, fluorescence was measured at 485 and 530 nm using the Varioskan LUX microplate reader.

The level of intracellular ROS was calculated using the same equation as mentioned above for cell viability calculations.

### 2.6. Statistical Analysis

The data from three independent experiments performed for all assays were analyzed using Statistica 13.3 PL software (StatSoft Poland Sp. z o.o., Krakow, Poland). The results are presented as the mean value ± standard deviation (SD). The normality of the distribution was examined using the Shapiro–Wilk test, and as each of the systems met the assumptions, parametric tests were subsequently applied. To assess differences between groups, analysis of variance (ANOVA) followed by post hoc Tukey’s test was used, and statistical significance was considered at *p*-values below 0.05, denoting a threshold for significance.

## 3. Results

### 3.1. Effect of Tested Compounds and Their Combinations on the Viability of PC12 Cells

**Figure 3:**
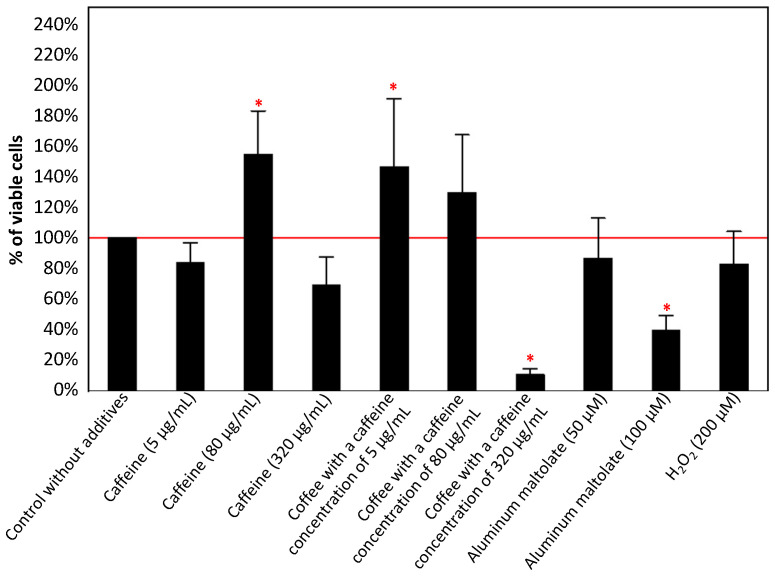
Effect of caffeine (5, 80, and 320 µg/mL), coffee (caffeine concentration of 5, 80, and 320 µg/mL), aluminum maltolate (50 µM and 100 µM), and H_2_0_2_ (200 µM) on the viability of PC12 cells after 24 h incubation in comparison to the control cells without additives. The results are presented as mean ± standard deviation (SD) (indicated as the upper edge of the column and whiskers, respectively) of three independent experiments. The cut-off point is marked with a red line. The significance of the differences was determined using the post hoc Tukey’s test. * *p* < 0.05.

**Figure 4:**
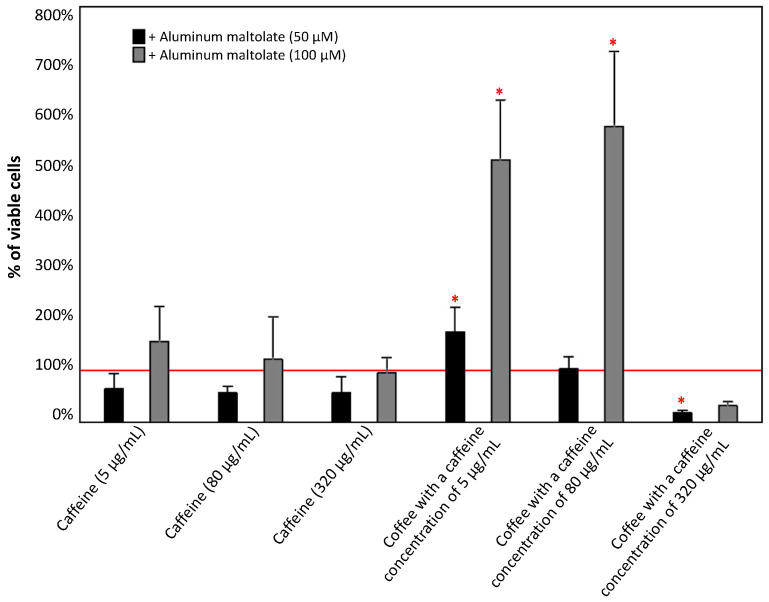
Effect of caffeine (5, 80, and 320 µg/mL) and coffee (caffeine concentration of 5, 80, and 320 µg/mL) on the viability of PC12 cells after 24 h incubation in comparison to the control cells cultured with 50 µM and 100 µM aluminum maltolate. The results are presented as the mean ± standard deviation (SD) (indicated as the upper edge of the column and whiskers, respectively) of three independent experiments. The cut-off point is marked with a red line. The significance of the differences was determined using the post hoc Tukey’s test. * *p* < 0.05.

**Figure 5:**
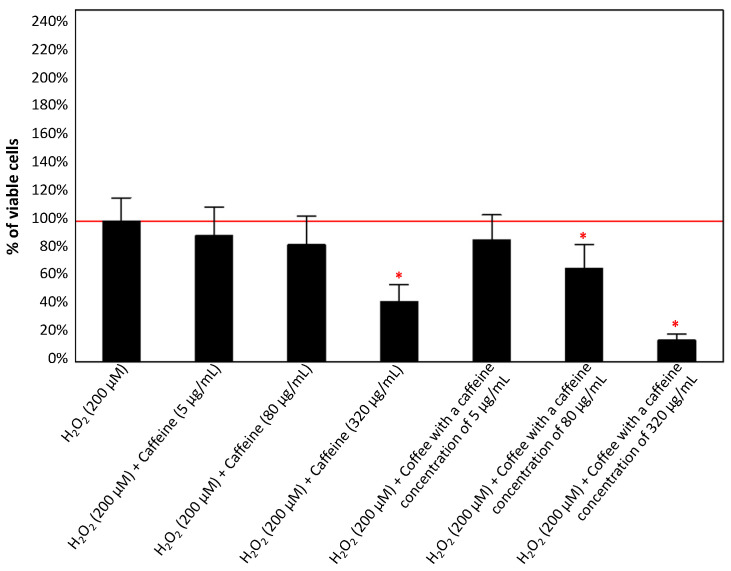
Effect of caffeine (5, 80, and 320 µg/mL) and coffee (with a caffeine concentration of 5, 80, and 320 µg/mL) on the viability of PC12 cells after 24 h incubation in comparison to the control cells cultured with H_2_O_2_ (200 µM). The results are presented as the mean ± standard deviation (SD) (indicated as the upper edge of the column and whiskers, respectively) of three independent experiments. The cut-off point is marked with a red line. The significance of the differences was determined using the post hoc Tukey’s test. * *p* < 0.05.

**Figure 6:**
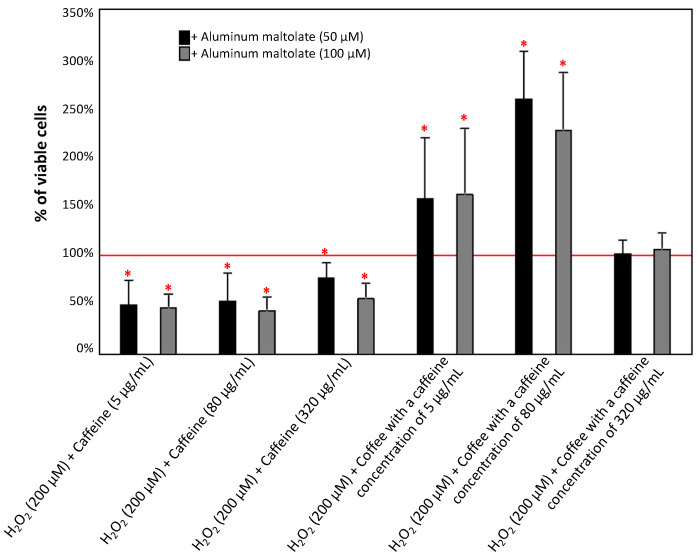
Effect of aluminum maltolate (50 µM and 100 µM) on the viability of PC12 cells after 24 h incubation in comparison to the control cells cultured with H_2_O_2_ (200 µM) and caffeine or caffeinated coffee. The results are presented as the mean ± standard deviation (SD) (indicated as the upper edge of the column and whiskers, respectively) of three independent experiments. The cut-off point is marked with a red line. The significance of the differences was determined using the post hoc Tukey’s test. * *p* < 0.05.

PC12 cells were treated individually with caffeine (5, 80, and 320 µg/mL), coffee (caffeine concentration of 5, 80, and 320 µg/mL), Almal (50 µM and 100 µM), H_2_0_2_ (200 µM), or with selected combinations of these compounds for 24 h. Cell viability was assessed using the MTT assay. The results are presented in Figure 3, Figure 4, Figure 5 and Figure 6.

#### 3.1.1. Effect of the Single Substances

As shown in Figure 3, caffeine at a concentration of 80 µg/mL and coffee containing 5 µg/mL caffeine significantly increased PC12 cell viability by 54% and 46%, respectively. A significant cytotoxic effect was caused by coffee containing 320 µg/mL of caffeine and 100 µM Almal (about 89% and 60%, respectively).

#### 3.1.2. Effect of the Combinations of Caffeine or Coffee Containing Caffeine with Aluminum Maltolate

Figure 4 shows the effect of combinations of Almal at 50 µM and 100 µM with caffeine or caffeinated coffee on cell viability. All combinations of Almal (50 µM) with different concentrations of caffeine caused a non-significant decrease in cell viability compared to the effect of Almal alone. However, the same combinations with 100 µM Almal had no effect or led to a non-significant increase in cell viability.

The combination of Almal (50 µM) with caffeinated coffee (with 5 ug/mL of caffeine) demonstrated a significant increase in cell viability of about 74% compared to Almal alone. A significant decrease in PC12 cell viability was observed when 50 µM Almal was combined with coffee containing 320 µg/mL of caffeine. In the case of PC12 cells cultured with Almal at a concentration of 100 µM, coffee with caffeine concentrations of 5 and 80 µg/mL increased cell viability by more than fivefold.

#### 3.1.3. Effect of Caffeine or Coffee Containing Caffeine on Cells Exposed to H_2_O_2_ without or with the Addition of Aluminum Maltolate

Figure 5 shows cell viability after PC12 cell treatment with H_2_O_2_ and caffeine or caffeinated coffee. All tested concentrations of both substances in combination decrease cell viability compared to H_2_O_2_ alone of 11–85%, depending on the caffeine concentration.

As presented in Figure 6, the exposure of PC12 cells to a combination of Almal, H_2_O_2_, and caffeine resulted in a decrease in the viability of cells of 22–50% and of 43–53% compared to Almal at 50 µM and 100 µM, respectively. However, the combination of Almal, H_2_O_2_, and coffee containing 5 and 80 µg/mL of caffeine caused an increase in the viability of cells of 58% to 159%, depending on the concentration of Almal and caffeine.

### 3.2. Effect of Tested Compounds and Their Combinations on ROS Generation

The level of intracellular ROS production in each configuration was assessed using the DCF-DA assay.

#### 3.2.1. Effect of the Single Substances

**Figure 7:**
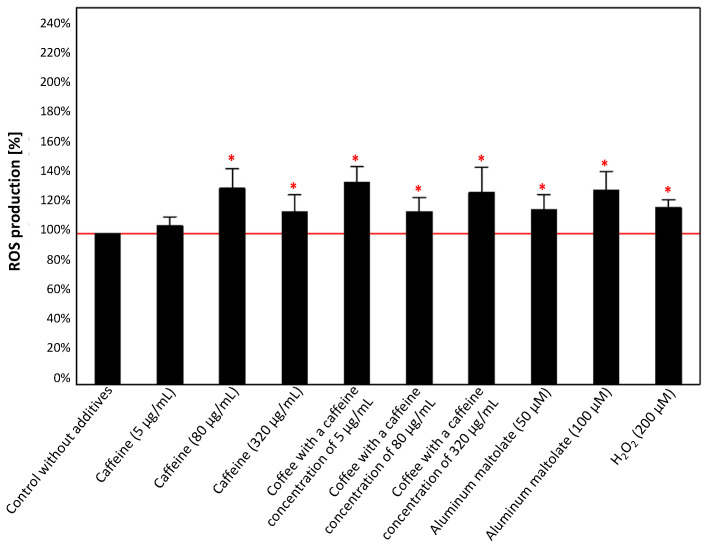
Effect of caffeine (5, 80, and 320 µg/mL), coffee (caffeine concentration of 5, 80, and 320 µg/mL), aluminum maltolate (50 µM and 100 µM), and H_2_0_2_ (200 µM) on ROS production in the culture of PC12 cells after 24 h incubation in comparison to the control cells without additives. The results are presented as the mean ± standard deviation (SD) (indicated as the upper edge of the column and whiskers, respectively) of three independent experiments. The cut-off point is marked with a red line. The significance of the differences was determined using the post hoc Tukey’s test. * *p* < 0.05. ROS—reactive oxygen species.

The effect of individual substances, i.e., caffeine (5, 80, and 320 µg/mL), coffee (caffeine concentration of 5, 80, and 320 µg/mL), Almal (50 µM and 100 µM), and H_2_O_2_ (200 µM), is shown in Figure 7. All tested substances caused a similar effect on ROS production in PC12 cells, inducing increases in intracellular ROS level of 15–34%. The effect was significant, except for the configuration with 5 µg/mL caffeine.

#### 3.2.2. Effect of the Combinations of Caffeine or Coffee Containing Caffeine with Aluminum Maltolate

**Figure 8:**
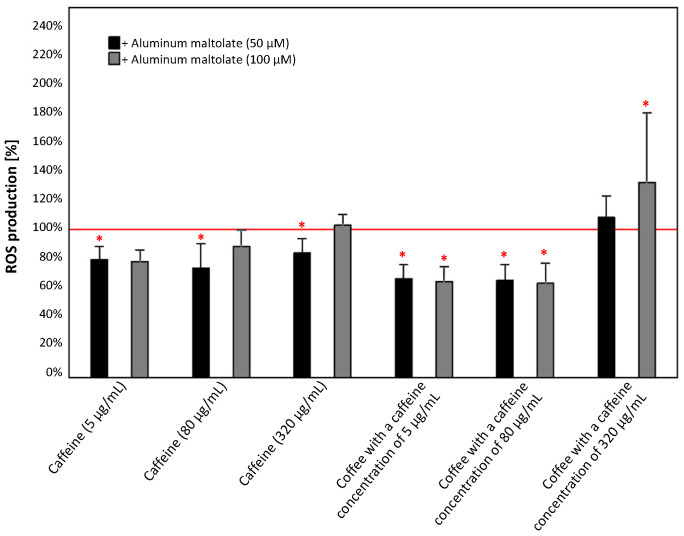
Effect of caffeine (5, 80, and 320 µg/mL) and coffee (caffeine concentration of 5, 80, and 320 µg/mL) on t ROS production in the culture of PC12 cells after 24 h incubation in comparison to the control cells cultured with 50 µM and 100 µM aluminum maltolate. The results are presented as the mean ± standard deviation (SD) (indicated as the upper edge of the column and whiskers, respectively) of three independent experiments. The cut-off point is marked with a red line. The significance of the differences was determined using the post hoc Tukey’s test. * *p* < 0.05. ROS—reactive oxygen species.

As shown in Figure 8, the combination of 50 µM Almal with caffeine and caffeinated coffee (except for that with a caffeine concentration of 320 µg/mL) leads to a significant reduction in ROS production of 16–34% compared to the control (50 µM Almal). A similar effect was observed for the combination with 100 µM Almal; however, this effect was significant only for caffeinated coffee. These combinations reduced the level of intracellular ROS in PC12 cells by 35% (for coffee containing 5 and 80 µg/mL of caffeine), while at a higher caffeine concentration, it increased it by 32%.

#### 3.2.3. Effect of Caffeine or Coffee Containing Caffeine on Cells Exposed to H_2_O_2_ without or with the Addition of Aluminum Maltolate

**Figure 9:**
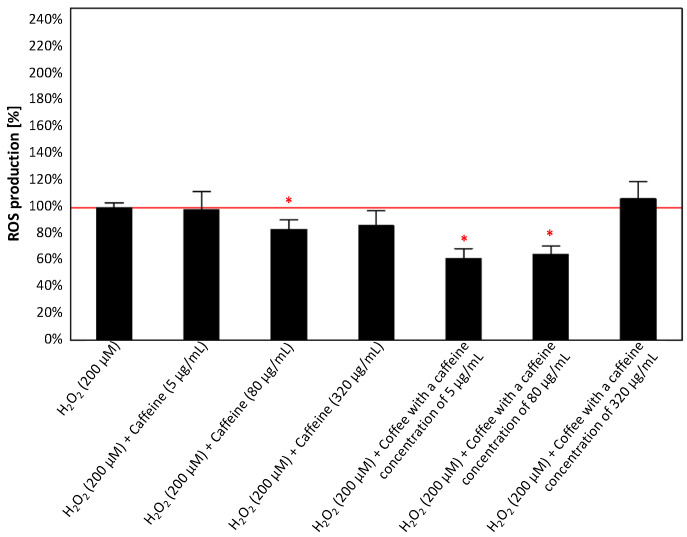
Effects of caffeine (5, 80, and 320 µg/mL) and coffee (with a caffeine concentration of 5, 80, and 320 µg/mL) on ROS production in the culture of PC12 cells after 24 h incubation in comparison to the control cells cultured with H_2_O_2_ (200 µM). The results are presented as the mean ± standard deviation (SD) (indicated as the upper edge of the column and whiskers, respectively) of three independent experiments. The cut-off point is marked with a red line. The significance of the differences was determined using the post hoc Tukey’s test. * *p* < 0.05. ROS—reactive oxygen species.

As shown in Figure 9, both caffeine and caffeinated coffee reduced the intracellular ROS level compared to control cells exposed to H_2_O_2_ (except for the configuration with coffee containing 320 µg/mL of caffeine). However, the effect was significant for 80 µg/mL caffeine and coffee with caffeine concentrations of 5 and 80 µg/mL. For these experimental conditions, a reduction of 16–38% was noted compared to the control (200 µM H_2_O_2_).

**Figure 10:**
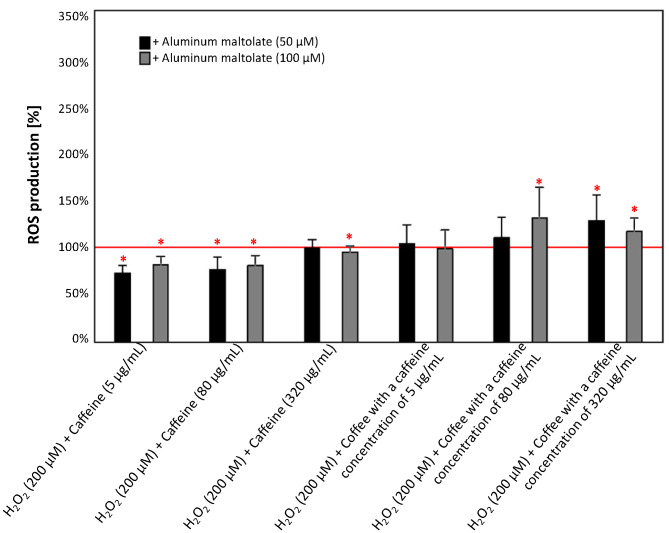
Effects of aluminum maltolate (50 µM and 100 µM) on the ROS production in the culture of PC12 cells after 24 h incubation in comparison to the control cells cultured with H_2_O_2_ (200 µM) and caffeine or caffeinated coffee. The results are presented as the mean ± standard deviation (SD) (indicated as the upper edge of the column and whiskers, respectively) of three independent experiments. The cut-off point is marked with a red line. The significance of the differences was determined using the post hoc Tukey’s test. * *p* < 0.05. ROS—reactive oxygen species.

As illustrated in Figure 10, the addition of Almal at both concentrations to configurations containing H_2_O_2_ and caffeine resulted in a decrease in ROS production (except for 50 µM aluminum maltolate in the configuration with H_2_O_2_ and 320 ug/mL caffeine) of 5–27%. However, the addition of 50 µM Almal to the configuration containing H_2_O_2_ and caffeinated coffee with 320 µg/mL caffeine increased ROS production by 17%. The addition of 100 µM Almal elevated ROS production in configurations containing H_2_O_2_ and caffeinated coffee with 80 and 320 µg/mL caffeine by 31% and 17%, respectively.

## 4. Discussion

Neurodegenerative diseases for years have been the subject of scientific interest from researchers worldwide due to the unclear mechanisms leading to their development. Recently, the neurotoxic effect of numerous environmental factors has been demonstrated [47,48]. One of them is exposure to aluminum and its compounds. Researchers are also focusing on oxidative stress, the consequences of which may be associated with the dysfunction of practically every part of the human body, including the nervous system. To better understand the mechanisms underlying neurotoxicity and neurodegenerative diseases, various experimental models have been developed in recent years based on experiments conducted on cell lines. One of the most commonly used cell lines is PC12. PC12 cells derived from a rat pheochromocytoma have undergone thorough characterization for their neurosecretory capabilities (i.e., the secretion of catecholamines, dopamine, and norepinephrine), and, for this reason, they are extensively used in in vitro studies related to neurodegenerative diseases [49]. With the expansion of knowledge regarding the negative impact of various environmental factors contributing to the onset of these diseases, questions have arisen about how to prevent the actions of these factors. One promising direction of research appears to be the effect of caffeinated coffee and its compounds [37]. In the present study, an experimental model was designed to explore the impact of caffeinated coffee and caffeine on the survival of PC12 cells and the generation of ROS when exposed to neurotoxic agents, such as Almal and/or hydrogen peroxide. ROS production is also closely related to the Fenton reaction. Within the framework of the experiment investigating the toxic impact of H_2_O_2_ on PC12 cells, the analysis of the Fenton reaction may be significant for the accurate interpretation of results obtained. H_2_O_2_ acts as a key component in the Fenton reaction and is also a substrate for the production of highly reactive hydroxyl free radicals (^•^OH) formed in the presence of transition metal ions, mainly ferrous (Fe^2+^) ions [26]. Furthermore, Al can enhance the Fenton reaction in the presence of iron ions [27,50]. Consequently, this reaction may engender escalated cellular damage and cell death [27]. The compounds inherent in caffeinated coffee have antioxidative properties; thus, free oxygen radicals generated in the Fenton reaction might be neutralized [51]. However, the aim of the present study was not focused on analyzing the Fenton reaction course but on researching whether caffeinated coffee, with all its ingredients, affects PC12 cells exposed to various configurations of tested substances, according to the procedure presented schematically in Figure 2.

The initial phase involved assessing the independent effect of each tested compound on PC12 cell survival and ROS production in cultures, considering different concentrations of caffeine (either alone or in coffee), Almal, and hydrogen peroxide. In the presence of caffeine at a concentration of 80 µg/mL and coffee with a caffeine concentration of 5 µg/mL, cell viability significantly increased. However, despite the lack of statistical significance, an increase in cell viability was also observed in the configuration with coffee at a caffeine concentration of 80 µg/mL. In the remaining configurations, a decrease in cell viability was observed, particularly in the configuration with coffee at a caffeine concentration of 320 µg/mL and Almal at a concentration of 100 µM. In each configuration (except for 5 µg/mL of caffeine), an increase in ROS production was observed, which might elucidate the adverse effect of higher concentrations of the investigated substances on PC12 cells. In reference to caffeine, the presented observations align closely with those documented in the literature. Yang et al. [44] demonstrated that lower concentrations of caffeine (5, 20, and 80 µg/mL) marginally enhance the viability of PC12 cells compared to the control, whereas a concentration of 320 µg/mL leads to a significant decrease in cell viability. The results presented here also partially line up with those obtained by Chian et al. [45] and demonstrated that caffeine reduces the viability of PC12 cells depending on the dosage. The authors used different caffeine concentrations (0.5 mM, 1 mM, 2 mM, and 3 mM), which, after taking into account the molar mass of caffeine of 194.19 g/mol, can be easily converted to approximately 97 µg/mL, 194 µg/mL, 388 µg/mL, and 582 µg/mL, respectively. The observed increase in PC12 cell viability in the present study occurred at a caffeine concentration below the point where Chian et al. [45] initiated their investigations; however, our observation regarding higher caffeine concentrations aligns with the findings of the authors. One of the proposed causes of such changes is apoptosis induced by an increase in ROS levels, which was also observed in the discussed experiment [45]. In the present study, a similar trend in the case of caffeinated coffee was observed, as an increase in caffeine concentration was associated with a decrease in the viability of PC12 cells. Furthermore, we also showed that exposure of PC12 cells to 100 µM Almal led to several PC12 cell damage events. Wang et al. [52] indicated that Almal induces a time- and dose-dependent decrease in PC12 cell viability, which was significant at a concentration of 100 µM. Our results are in accordance with those obtained by Wang et al. [52] and confirm the neurotoxicity of the selected Al compound. Additionally, Yu et al. [53] also documented that the utilization of Al in the form of maltolate is more accurate and effective in inducing neurotoxicity compared to other Al compounds, such as aluminum chloride. The authors demonstrated that Almal at concentrations of 0.5 mM and 3.0 mM induces augmented ROS production in PC12 cells, potentially leading to cell apoptosis, and their research results suggest that Almal even at the concentration of 0.1 mM significantly enhances ROS production. Increased ROS production in response to Almal might be a factor leading to Al neurotoxicity. The observations of Yu et al. [53] are in accordance with study results obtained previously by Cheng et al. [54] who demonstrated that Al triggers ferroptosis through the activation of oxidative damage signaling pathways in neurons, leading to cell damage. Moreover, Xu et al. [55] demonstrated that aberrant activation of the 3-kinase (PI3K)/Akt/mammalian target of rapamycin (mTOR) (PI3K/Akt/mTOR) signaling pathway by Almal may contribute to diminished synaptic plasticity and impaired cognitive function. This observation implies that the PI3K/Akt/mTOR signaling cascade likely plays a comparable role to Al in the induction of cognitive dysfunction, substantiating the notion that Al is a significant environmental factor associated with neurodegenerative diseases [55]. Xu et al. [55] also reported that the activated PI3K/Akt/mTOR pathway, together with reduced expression of synaptic plasticity-related proteins (PRPs) and heightened p-tau deposition, could potentially contribute to Al-induced neurotoxicity. In the past, many other potential mechanisms that may explain Al activity as a factor inducing neurotoxicity (reviewed by Skalny et al. [16]) were proposed. However, the regulation of the mitogen-activated protein kinase (MAPK) signaling pathway, as discussed in detail in the following paragraphs, might be a crucial determinant in this process [56]. In our study the decrease in cell viability, although not significant, was observed when hydrogen peroxide was used. The selected H_2_O_2_ concentration may not have been sufficiently high to elicit significant effects, but it can be assumed that toxicity can be induced by 200 µM hydrogen peroxide similarly as it was reported by other authors [57,58,59,60]. Thus, the present study demonstrates that caffeine and caffeinated coffee can exert both positive and negative effects on cell survival, depending on the caffeine concentration. In contrast, Almal and hydrogen peroxide reduce cell survival, suggesting the neurotoxic effect of these substances, and consequently, their potential contribution to the development of neurodegenerative diseases.

The results of epidemiological studies suggest that the consumption of caffeinated coffee reduces the risk of developing neurodegenerative diseases [37,38]. In the era of common knowledge about the negative impact of various environmental factors (such as excessive exposure to Al compounds) on the nervous system and their association with increased risk of neurotoxicity and consequently neurodegenerative diseases, the role of natural substances commonly used in daily diet becomes crucial. In the present study, it was noted that caffeine alone does not enhance the viability of PC12 cells when they were exposed to 50 µM Almal. However, we observed a positive effect when caffeinated coffee was used, as an increase in cell survival was noticeable at a caffeine concentration of 5 µg/mL. Even though in almost every case, with one exception for coffee with a caffeine concentration of 320 µg/mL, the combination of 50 µM Almal with caffeine/caffeinated coffee caused a reduction in ROS production, thereby demonstrating the antioxidant properties of caffeine and coffee in response to the action of Almal, it is unlikely that this is a direct effect and the only mechanism of protective action of coffee because (however, not in all cases) it did not translate into an increase in the survival of PC12 cells. It should be also noted that the application of 50 µM Almal did not significantly decrease cell survival despite a significant increase in ROS levels, making it difficult to assert a protective effect of caffeinated coffee in this case. On the other hand, when the concentration of Almal was higher (100 µM) and induced a significant decrease in the viability of PC12 cells, the observed effect of coffee action became more pronounced. Caffeine alone at concentrations of 5 µg/mL and 80 µg/mL in combination with 100 µM Almal caused a slight increase in PC12 cell survival, and coffee with a caffeine concentration of 5 µg/mL and 80 µg/mL increased cell survival even up to fivefold compared to 100 µM Almal alone. In the discussed configurations, we also observed a reduction in ROS production, which suggests that the antioxidant action of components of caffeinated coffee might play a role in preventing cell death. It was previously documented that higher concentrations of caffeine (both alone and in coffee) induce cell death in cell cultures exposed to 100 µM Almal, which is justified by the toxicity of caffeine at high doses [45]. In this context, changes in ROS production also may play an important role, as the ROS levels in these configurations are higher than those observed with 100 µM Almal alone. Interestingly, the observed positive effects are particularly evident in the case of caffeinated coffee, rather than for caffeine alone, suggesting that other components of coffee may be responsible for protecting cells from the toxic activity of Almal. These observations are partially in accordance with study results obtained by Yang et al. [44] who investigated the influence of caffeine on toxicity induced by compounds of metals other than Al and did not demonstrate that caffeine significantly reduced the percentage of apoptotic PC12 cells. On the other hand, Hosny et al. [61] in their study using an animal model observed that caffeine confers notable neuroprotection against AlCl_3_-induced neurotoxicity, primarily through its antioxidant, anti-inflammatory, and anticholinesterase properties. The above indications suggest that caffeine in the body may have a different effect than in cellular models, and a potential reason for this could be the complex metabolism of caffeine in the human liver, leading to the formation of various metabolites capable of exerting different effects in the body [33].

**Figure 11:**
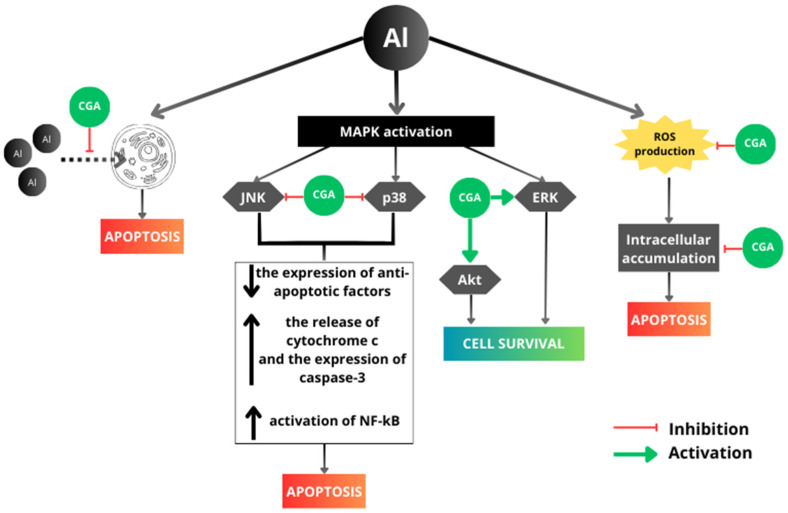
Potential mechanisms of aluminum toxicity and the protective action of CGA. Al—aluminum, CGA—chlorogenic acid, ERK—extracellular signal-regulated kinase, JNK—c-Jun amino-terminal kinase, MAPK—mitogen-activated protein kinase, NF-κB—nuclear factor kappa B, ROS—reactive oxygen species. Black arrows: ↑—increase, ↓—decrease.

In coffee, a variety of other substances can be found, among them polyphenols, with chlorogenic acid (CGA) being a prominent representative [62]. As it is known, the highest content of phenolic compounds is exhibited by instant coffee [63]. CGA possesses antioxidant, anti-inflammatory, and many other biological activities [64,65]. Study results offer compelling evidence supporting the positive effects of CGA in the context of its activity during the development of neurodegenerative diseases because of its ability to penetrate the blood–brain barrier, thereby exhibiting neuroprotective effects in brain tissue [66,67]. In the context of metal toxicity, the properties of polyphenols as metal-chelating agents appear significant [68]. For instance, some authors have demonstrated that the utilization of polyphenols can diminish the absorption of Al into the gastrointestinal tract through its chelation [69]. Hence, the chelation of CGA with metals might emerge as a crucial mechanism for alleviating the neurotoxicity associated with aluminum. Cheng et al. [70] examined the protective impact of CGA on the apoptosis of PC12 cells induced by AlCl_3_. The authors observed a significant enhancement in the viability of PC12 cells and a reduction in the apoptotic rate under various intervention conditions when CGA was used. The most pronounced alleviation effect was observed when the Al compound and CGA were simultaneously administered. These findings might elucidate why, in the present experiment, a substantial increase in the viability of PC12 cells was observed when caffeinated coffee, rich in CGA, was used. A potential protective mechanism of CGA against Al toxicity, also proposed by Cheng et al. [70], may involve Al chelation. This process induces an increase in the molecular mass of aluminum, consequently reducing the intensity and rate of Al penetration into cells, which, in turn, translates to enhanced viability of PC12 cells. Such a mechanism appears highly plausible, as increased Al accumulation in cells leads to apoptosis [69]. On the other hand, the regulation of the MAPK pathway may also play a significant role. This pathway is composed of three kinase modules that are activated sequentially through phosphorylation by their respective upstream kinases [71]. Eukaryotic cells are equipped with numerous MAPK pathways that collaboratively govern processes such as gene expression, mitosis, cell metabolism, motility, survival, apoptosis, and differentiation [72]. The most widely known are the conventional MAPK pathways, comprising the extracellular signal-regulated kinase (ERK), c-Jun amino-terminal kinase (JNK), and p38 MAPK families, which may be activated by mitogens, cytokines, and cellular stress [72]. Activation of JNK and p38 MAPK, especially when coupled with concurrent inhibition of ERK, is crucial for inducing apoptosis in cells [73]. This reaction is associated with the expression of apoptosis-related proteins, as the activation of JNK and p38 MAPK is related to the inhibition of antiapoptotic protein expression from the BCL-2 family, particularly BCL-X_L_, and the release of cytochrome c and the subsequent activation of expression of caspase-3, which plays a crucial role in the apoptosis process [74]. On the other hand, activation of the MAPK pathway, especially ERK, can lead to the stimulation of the cell proliferation process, thereby influencing cell survival and protecting them from apoptosis [72]. Among the factors influencing the activation of the MAPK pathway, aluminum can be identified. Studies on the brains of rats exposed to Al have shown that Al led to a slight increase in ERK activity and significant activation of p38 MAPK, which may induce neurotoxicity and consequently lead to the development of neurodegenerative diseases [56]. It is worth noting that activation of p38 MAPK leads to the hyperphosphorylation of tau protein, known for its role in the pathogenesis of Alzheimer’s disease [75]. Moreover, persistent activation of the JNK or p38 MAPK signaling pathways has been proposed to mediate neuronal apoptosis in AD and PD [76]. Coffee components, specifically CGA, may impact MAPK pathways. Cho et al. [74] documented that the inhibitory effect of decaffeinated coffee and CGA on H_2_O_2_-induced apoptosis in PC12 cells involves the reduction of JNK and p38 MAPK activation. The authors also observed that preincubation of PC12 cells with decaffeinated coffee and CGA attenuates H_2_O_2_-induced apoptosis by blocking the downregulation of Bcl-X_L_ and caspase-3 expression in PC12 cells. Cheng et al. [77] reported that exposure of RAW264.7 macrophages to Al compound increases the expression of JNK and decreases the expression of ERK, leading to cell apoptosis. The authors indicated that CGA reverses these changes by inducing an increase in ERK and a decrease in JNK activation, thereby enhancing cell survival. Moreover, Cheng et al. [77] observed that CGA also inhibited the expression of pro-apoptotic factors (a pro-apoptotic Bcl-2 family protein (Bad) and cytochrome c), which increased in response to aluminum chloride. A significant discovery was also the observed increase in the expression of Akt kinase protein (regulating cell viability and growth) induced by CGA, which was confirmed in a model similar to that presented in this study, based on the action of aluminum chloride on PC12 cells [70]. Finally, another crucial factor examined by other scientists is nuclear factor kappa B (NF-κB), which is a critical component of intracellular signaling pathways involved in inflammatory processes. Its activation increases along with the activation of MAPK (Al activates MAPK, as it was already mentioned in this paragraph) and leads to an elevation in the production of pro-inflammatory factors, potentially causing cell damage [78]. As indicated by the latest research, CGA inhibits NF-κB by suppressing p38 MAPK activation, contributing to the prevention of cell death [79,80,81]. It can be concluded that constituents of caffeinated coffee, likely primarily CGA, mitigate the neurotoxicity of Al and thus reduce the risk of neurodegenerative diseases development. The potential mechanisms through which CGA may influence the survival of PC12 cells exposed to the toxic effect of Al are shown in Figure 11.

Numerous authors have highlighted the antioxidative properties of caffeine and caffeinated coffee [31,33,67]. In the current study, caffeine and caffeinated coffee caused a decrease in ROS levels in PC12 cell culture medium when the factor inducing the free radical cascade was H_2_O_2_. However, not only did this not lead to an increase in PC12 cell survival, but, on the contrary, cells treated with all configurations of hydrogen peroxide and caffeine/caffeinated coffee exhibited higher mortality than in the case of hydrogen peroxide action alone. It follows that despite their antioxidant properties, caffeine and caffeinated coffee do not contribute to increased cell viability by mitigating oxidative stress. Additionally, as it was reported by Pavlica et al. [82], the decrease in ROS levels is not a direct cause of higher PC12 cell viability. On the other hand, Kim et al. [83] in their study on primary cortical neurons demonstrated that pretreatment with both caffeinated and decaffeinated coffee inhibited H_2_O_2_-induced apoptotic neuronal death through inhibition of the H_2_O_2_-induced decrease in the expression of antiapoptotic proteins Bcl-2, especially Bcl-X_L_, and the prevention of H_2_O_2_-induced pro-apoptotic cleavage of caspase-3 and pro-poly(ADP-ribose) polymerase (pro-PARP). Moreover, the authors observed that both caffeinated and decaffeinated coffee, as well as CGA, induced the expression of NADPH:quinone oxidoreductase 1 (NQO1) in neuronal cells, indicating that these substances safeguard neurons from H_2_O_2_-induced apoptosis through the upregulation of this antioxidant enzyme. Additionally, Cho et al. [74] demonstrated that preincubation with decaffeinated coffee and CGA prevents H_2_O_2_-induced toxicity by blocking both the accumulation of intracellular ROS and the activation of p38 MAPK. The authors used 200 μM H_2_O_2_, which is also used in our study. On the other hand, Yao et al. [84] demonstrated that pretreatment of PC12 cells with CGA offered neuroprotection against H_2_O_2_-induced toxicity by directly neutralizing free radicals and indirectly by triggering the endogenous antioxidant enzymes via the activation of nuclear factor erythroid 2–related factor 2 (Nrf2). A detail that might explain discrepancies between the results of the present study and those obtained by other authors is that in our experiments cells were not preincubated with caffeine or caffeinated coffee without any other additives, but they were incubated with a mixture of caffeine/caffeinated coffee and H_2_O_2_. It is possible that to enhance cell survival in response to H_2_O_2_ toxicity, the prior activation of cellular defense mechanisms is necessary. What is particularly noteworthy is the multitude of potential mechanisms protecting cells against oxidative stress, which indicate the versatile positive effects of certain components of coffee.

In the subsequent experiment, another objective was to confirm the hypothesis that Almal intensifies oxidative stress provoked by hydrogen peroxide. We anticipated that the combination of two components, hydrogen peroxide and Almal, would result in increased toxicity of these components, which caffeine or caffeinated coffee may not be able to mitigate, or differences would not be significant. Indeed, such an outcome occurred when cells were exposed to H_2_O_2_ and caffeine; moreover, simultaneous addition of Almal at both examined concentrations caused even greater mortality of PC12 cells than in the case of separate exposure to H_2_O_2_ and caffeine despite a reduction in the level of ROS. However, some observations concerning caffeinated coffee action turned out to be rather surprising. In configurations with H_2_O_2_ and coffee at caffeine concentrations of 5 µg/mL and 80 µg/mL, the simultaneous addition of Almal at both tested concentrations resulted in an increase in cell survival (even despite the elevation in ROS levels in the configuration with H_2_O_2_ and coffee with a caffeine concentration of 80 µg/mL and 100 µM Almal), leading to PC12 cell survival levels comparable to the control culture without any additives. These results confirm earlier findings that there is at least one additional mechanism of the protective action of caffeinated coffee against the toxic effects of the examined compounds that differs from a reduction in ROS levels or chelation of Al by coffee components. Furthermore, it can be speculated that Almal activates certain cellular mechanisms, while coffee components inhibit those that would typically lead to cell apoptosis, as observed in PC12 cultures with Almal alone. As mentioned previously, Al affects the MAPK pathway, strongly activating p38 MAPK and slightly ERK, among others [56]. However, CGA found in coffee can reduce the activation of p38 MAPK and increase the activation of ERK, which may explain the increased cell survival [74,77]. A very interesting finding is that for CGA to exert its effects, Al must activate the MAPK pathway, which seems to confirm the observed significant increase in cell survival when Almal was added.

Based on the results of the current study and information from the research results of other authors, it can be concluded that the key component of coffee that protects cells against unfavorable changes caused by Al compounds is not caffeine, but most likely CGA. The mechanisms by which this compound has a positive effect on cell survival are multidirectional, with many aspects, and require further research to identify molecular targets for which modification could prevent the neurotoxicity of Al compounds and, consequently, the development of neurodegenerative diseases caused by them. It is also highly probable that inhibiting some MAPK pathways, and/or promoting others, could result in the effective prevention of these diseases. Moreover, it can be hypothesized that people particularly exposed to Al may benefit most from the protective effects of caffeinated instant coffee in the context of preventing/neutralizing the neurotoxicity of this element because the coffee they consume may affect cell signaling pathways leading to the inhibition of cell apoptosis and activation of their proliferation. However, we should be careful when drawing conclusions and formulating hypotheses because the analyzed results were obtained in in vitro studies using a cell line. The real consequences of consuming caffeinated coffee in the context of exposure to Al compounds most probably are much more complex in the human body, e.g., due to individual differences. Therefore, further research, including both in vitro and clinical studies, is necessary to verify the hypotheses put forward in this study and to understand the full spectrum of the protective effect of caffeinated coffee against the neurotoxicity of Al compounds in humans.

In addition to the many interesting conclusions drawn from the presented research results, we must also mention that the conducted study has certain limitations related to the methods employed. While the PC12 cell line is commonly used in neurotoxicity studies, it should be emphasized that it originated from a rat adrenal pheochromocytoma tumor and biologically differs from primary neuronal cultures or human cell lines, potentially limiting the generalizability of the findings. Moreover, although the selection of concentrations of tested substances was based on information available in the literature, it should be kept in mind that optimal concentrations for in vitro studies can vary depending on cell type, experimental conditions, and assay sensitivity. Despite efforts to minimize interactions between tested substances with MTT and DCF-DA assays by rinsing the cells with PBS, it is important to recognize that residual compounds or byproducts of cell metabolism may still affect assay readouts. Some authors suggest that plant extracts and polyphenolic compounds (such as those occurring in coffee) may interfere with the MTT assay [85,86]. Another limitation is that the DCF-DA assay is non-specific and is sensitive to various factors that impact its reproducibility, such as esterase activity and the availability of Fe for the Fenton reaction [87].

## 5. Conclusions

Exposure to aluminum and its compounds is an environmental factor that induces neurotoxicity, partly through oxidative stress, ultimately leading to the development of neurodegenerative diseases. It has been demonstrated that components of the daily diet, such as caffeinated coffee, may play a significant role in preventing these diseases.

The present study illustrates that caffeinated coffee has the potential to prevent Almal-induced neurotoxicity in PC12 cells, confirming the neuroprotective effect of coffee containing appropriate concentrations of caffeine. Simultaneous treatment of cells with 100 μM Almal and coffee at caffeine concentrations of 5 μg/mL and 80 μg/mL resulted in a higher than fivefold increase in the survival rate of PC12 cells. In light of the absence of similar observations with caffeine alone, it can be inferred that other components of caffeinated coffee are responsible for its protective effects. A good candidate mentioned many times by other researchers is CGA—an antioxidant that is a significant component of coffee. However, despite the demonstrated antioxidant properties of caffeine and caffeinated coffee responsible for the reduction of ROS level, it is highly unlikely that they are the sole or primary factors contributing to cell protection against neurotoxicity induced by both oxidative stress and Al exposure, as reduced ROS production did not always correlate with increased PC12 cell survival.

Based on the research results reported by other authors, it seems very probable that one of the protective mechanisms may involve the chelation of Al by CGA, which consequently prevents Al from entering the cell and thereby prevents cell apoptosis. An innovative aspect of this study is the simultaneous treatment of PC12 cells with two factors inducing neurotoxicity (H_2_O_2_ and Almal), which reveals that coffee components such as CGA exert their significant protective effect only after activating cellular defense mechanisms in response to Al.

While the observed properties of caffeinated coffee are promising in the context of preventing the neurotoxicity induced by Al compounds leading consequently to the development of neurodegenerative diseases associated with Al exposure, it is crucial to approach these findings with caution. The results obtained are based on in vitro studies using cell lines, and translating these outcomes to the complex human organism may prove more intricate. Therefore, further research, encompassing both in vitro and clinical studies, is imperative to validate all hypotheses and fully comprehend the mechanism of the protective impact of caffeinated coffee and its compounds that lead to the prevention of neurotoxicity induced by Al compounds in humans.

## Data Availability

The raw data supporting the conclusions of this article will be made available by the authors on a reasonable request.
